# Níveis séricos de sCD40L, CCL3 e NT-proBNP em Pacientes Idosos Internados com Insuficiência Cardíaca

**DOI:** 10.36660/abc.20260035

**Published:** 2026-03-02

**Authors:** Maria da Gloria Cruvinel Horta, Marcus Carvalho Borin

**Affiliations:** 1 Santa Casa de Misericórdia de Belo Horizonte Belo Horizonte MG Brasil Santa Casa de Misericórdia de Belo Horizonte, Belo Horizonte, MG - Brasil; 2 UNIMED-BH Belo Horizonte MG Brasil UNIMED-BH, Belo Horizonte, MG - Brasil

**Keywords:** Insuficiência Cardíaca, Trombose Venosa, Biomarcadores

Os autores investigaram os níveis séricos de sCD40L (ligante solúvel de CD40), da quimiocina pró-inflamatória CCL3 e do pró-peptídeo natriurético tipo B N-terminal (NT-proBNP) em pacientes idosos internados com insuficiência cardíaca (IC). O estudo explorou se a triagem desses biomarcadores, individualmente ou em combinação, pode servir como ferramenta diagnóstica para IC associada à trombose venosa profunda. A presença de trombos foi determinada por meio de ultrassonografia venosa dos membros inferiores durante a internação hospitalar. Avaliando 200 pacientes divididos em grupos com e sem trombose, os pesquisadores constataram que os grupos apresentaram características semelhantes, com exceção dos níveis de lipoproteínas, que foram significativamente maiores no grupo com trombose. A análise combinada de NT-proBNP, sCD40L e CCL3 apresentou sensibilidade de 62,00% e especificidade de 91,00%. Essa análise conjunta demonstrou ser superior ao desempenho individual de cada biomarcador, utilizando limiares de 264,93 pg/mL para NT-proBNP, 7,07 ng/mL para sCD40L e 28,96 ng/L para CCL3, destacando, assim, o valor diagnóstico da combinação para esse perfil específico de paciente.^1^

A relevância desses achados é corroborada pela literatura existente, como o trabalho publicado por Haller et al.,^2^ que investigou os desfechos prognósticos incrementais do NT-proBNP em conjunto com outros biomarcadores para estratificação de risco em pacientes com fibrilação atrial. Seus achados sugeriram que o uso futuro de múltiplos biomarcadores poderia auxiliar na distinção entre níveis de risco mais altos e mais baixos em pacientes cardíacos complexos. De forma similar, Folsom et al.^3^ conduziram um acompanhamento longitudinal de seis anos por meio do estudo "*Atherosclerosis Risk in Communities*" (Risco de Aterosclerose em Comunidades), avaliando medições seriadas de NT-proBNP. Seus dados mostraram que pacientes com aumento de NT-proBNP de 100 para ≥100 pg/mL apresentaram um risco 1,4 vezes maior de tromboembolismo venoso em comparação com aqueles com concentrações estáveis e mais baixas. Quando os níveis permaneceram consistentemente ≥100 pg/mL, o risco aumentou para 1,6 vezes. Isso sugere que o aumento dos níveis de NT-proBNP serve como um indicador de vulnerabilidade cardíaca iminente, predispondo os pacientes a eventos tromboembólicos.^3^

No entanto, a inclusão de biomarcadores plaquetários como o sCD40L adiciona uma camada de complexidade. Embora se saiba que o sCD40L aumenta a estabilidade do trombo e promove a expressão do fator tecidual e de moléculas pró-inflamatórias, sua aplicação clínica permanece controversa. Um estudo populacional com quase 3.000 indivíduos não conseguiu determinar uma correlação significativa entre os níveis de sCD40L e os fatores de risco para aterosclerose.^4^ Além disso, Burdess et al.^5^ criticaram o uso do sCD40L devido à falta de consistência nas medições realizadas no mesmo dia ou em dias diferentes, encontrando pouca correlação com os resultados da citometria de fluxo. Esses achados foram corroborados por Tsakiris et al.^6^ e Blann et al.^7^ em estudos sobre doença arterial periférica. Adicionalmente, Riedl et al.^8^ avaliaram marcadores de ativação plaquetária em pacientes com câncer e tromboembolismo, demonstrando que o sCD40L não foi capaz de predizer, de forma independente, o desenvolvimento de tromboembolismo venoso. Da mesma forma, o CCL3, embora associado a processos inflamatórios, permanece um biomarcador que ainda não foi completamente explorado em ensaios clínicos de grande escala.

Um biomarcador ideal deve ser específico, preciso e replicável por técnicas simples, além de ser custo-efetivo e aceitável para os pacientes. A identificação de alterações nos marcadores plasmáticos associadas a eventos tromboembólicos iminentes pode permitir ajustes terapêuticos oportunos e individualizados para prevenir a exacerbação da trombose. Apesar dos inúmeros estudos clínicos que avaliam biomarcadores no contexto da tromboembolismo venoso, os dados ainda são inconclusivos, indicando a necessidade de ensaios clínicos mais robustos para definir completamente seu potencial diagnóstico.

Em última análise, devemos aceitar e reconhecer a realidade dos sistemas de saúde em que atuamos para prevenir, diagnosticar e tratar eficazmente as complicações cardiovasculares. Embora as diretrizes clínicas muitas vezes descrevam um mundo utópico, impor esses padrões sem considerar as limitações sociais, econômicas e de acesso pode dificultar melhorias a curto prazo nos resultados dos pacientes. A investigação da combinação de sCD40L, CCL3 e NT-proBNP representa um esforço louvável para expandir os limites do diagnóstico, reconhecendo essas limitações sistêmicas. Essa abordagem enfatiza que a utilização de biomarcadores combinados pode desempenhar um papel significativo na prática clínica quando usada criteriosamente em conjunto com uma avaliação abrangente. Melhorar a fluidez da identificação e o encaminhamento oportuno é essencial para evitar situações em que os pacientes chegam ao sistema de saúde somente após sofrerem eventos agudos graves. Iniciativas que propõem estratégias viáveis, adaptadas às realidades da saúde pública, são vitais para abordar as disparidades no acesso à saúde. Se esperarmos por recursos infinitos para imitar os países de alta renda, não conseguiremos melhorar a qualidade de vida de nossos pacientes hoje; este estudo aponta para uma evolução necessária no uso da ciência acessível para fornecer o melhor tratamento disponível ao nosso alcance.
