# Supplementary material for: Níveis séricos de sCD40L, CCL3 e NT-proBNP em Pacientes Idosos Internados com Insuficiência Cardíaca
Source: Arq Bras Cardiol. 2026 Mar 2;123(1):e20260035. [Article in Portuguese] doi: 10.36660/abc.20260035 (PMC13127814; doi:10.36660/abc.20260035)
Supplement: Translation

# Translation: Serum Levels of sCD40L, CCL3, and NT-proBNP in Elderly Patients Admitted to the Hospital with HF

**DOI:** 10.36660/abc.20260035i

**Authors:** Maria da Gloria Cruvinel Horta, Marcus Carvalho Borin

**Affiliations:** 1 Belo Horizonte MG Brazil Santa Casa de Misericórdia de Belo Horizonte, Belo Horizonte, MG – Brazil; 2 Belo Horizonte MG Brazil UNIMED-BH, Belo Horizonte, MG – Brazil

**Keywords:** Heart Failure, Venous Thrombosis, Biomarkers

The authors investigated the serum levels of sCD40L (soluble CD40 Ligand), the pro-inflammatory chemokine CCL3, and N-terminal pro b-type natriuretic peptide (NT-proBNP) in elderly patients admitted to the hospital with HF. The study explored whether screening these biomarkers individually or in combination can serve as a diagnostic tool for HF associated with VTELL. The presence of thrombi was determined through venous ultrasound of the lower limbs during hospital admission. Evaluating 200 patients divided into groups with and without thrombosis, the researchers found that the groups presented similar characteristics, with the exception of lipoprotein levels, which were significantly higher in the thrombosis group. The combined analysis of NT-proBNP, sCD40L, and CCL3 yielded a sensitivity of 62.00% and a specificity of 91.00%. This associated analysis proved superior to the individual performance of each biomarker, utilizing thresholds of 264.93 pg/mL for NT-proBNP, 7.07 ng/mL for sCD40L, and 28.96 ng/L for CCL3, thereby highlighting the diagnostic value of the combination for this specific patient profile.^1^

The relevance of these findings is supported by existing literature, such as the work published by Haller et al.,^2^ which investigated incremental prognostic outcomes of NT-proBNP plus other biomarkers for risk stratification in patients with atrial fibrillation. Their findings suggested that future use of multiple biomarkers could help distinguish between higher and lower risk levels in complex cardiac patients. Similarly, Folsom et al.^3^ conducted a six-year longitudinal follow-up through the "Atherosclerosis Risk in Communities" study, evaluating serial measurements of NT-proBNP. Their data showed that patients with an NT-proBNP increase from 100 to ≥100 pg/mL presented a 1.4 times increased risk for venous thromboembolism compared to those with stable, lower concentrations. When levels were consistently ≥100 pg/mL, the risk rose to 1.6 times. This suggests that rising NT-proBNP levels serve as an indicator of imminent cardiac vulnerability, predisposing patients to thromboembolic events.^3^

However, the inclusion of platelet biomarkers like sCD40L adds a layer of complexity. While sCD40L is known to increase thrombus stability and promote the expression of tissue factor and pro-inflammatory molecules, its clinical application remains debated. A population study of nearly 3,000 subjects could not determine a significant correlation between sCD40L levels and atherosclerosis risk factors.^4^ Furthermore, Burdess et al.^5^ criticized the use of sCD40L due to a lack of consistency in measurements taken within the same day or between days, finding little correlation with Flow Cytometry results. These findings were echoed by Tsakiris et al.^6^ and Blann et al.^7^ in studies of peripheral arterial disease. Additionally, Riedl et al.^8^ assessed platelet activation markers in patients with cancer and thromboembolism, showing that sCD40L could not independently predict the development of VTE. Similarly, CCL3, while associated with inflammatory processes, remains a biomarker that has not yet been thoroughly explored in large-scale clinical trials.

An ideal biomarker should be specific, precise, and replicable by simple techniques, while remaining cost-effective and acceptable for patients. Identifying shifts in plasma markers associated with imminent thromboembolic events can allow for timely and individualized treatment adjustments to prevent the exacerbation of thrombosis. Despite the numerous clinical studies evaluating biomarkers in the context of VTE, data remain somewhat inconclusive, indicating the need for more robust trials to fully define their diagnostic potential.

Ultimately, we must embrace and recognize the reality of the healthcare systems in which we operate to effectively prevent, diagnose, and treat cardiovascular complications. While clinical guidelines often describe a utopian world, imposing these standards without considering social, economic, and accessibility limitations can hinder short-term improvements in patient outcomes. The investigation into the combination of sCD40L, CCL3, and NT-proBNP represents a laudable effort to push the boundaries of diagnosis by recognizing these systemic limitations. This approach emphasizes that utilizing combined biomarkers can provide a meaningful role in clinical practice when used judiciously alongside comprehensive assessment. Improving the fluidity of identification and timely referral is essential to avoid situations where patients reach the health system only after suffering severe acute events. Initiatives that propose feasible strategies tailored to public health realities are vital for addressing disparities in healthcare access. If we wait for infinite resources to imitate high-income countries, we will fail to improve the quality of life for our patients today; this study points toward a necessary evolution in using accessible science to provide the best available treatment within our reach.
